# Neck paraganglioma and follicular lymphoma: a case report

**DOI:** 10.1186/s13256-019-2323-1

**Published:** 2019-12-20

**Authors:** Lara Marchetti, Luca Perrucci, Francesca D’Ercole, Maria Chiara Zatelli, Maria Rosaria Ambrosio, Melchiore Giganti, Aldo Carnevale

**Affiliations:** 1 Department of Interventional and Diagnostic Radiology, Arcispedale Sant’Anna, Ferrara, Italy; 2 Section of Diagnostic Imaging, Department of Morphology, Surgery and Experimental Medicine, University of Ferrara, Ferrara, Italy; 3 University Radiology Unit, Radiology Department, Sant’Anna University Hospital, Ferrara, Italy; 4 Section of Endocrinology and Internal Medicine, Department of Medical Sciences, University of Ferrara, Ferrara, Italy

**Keywords:** Head and neck paraganglioma, Follicular lymphoma, SDH, Imaging

## Abstract

**Background:**

Paragangliomas and pheochromocytomas are sympathetic or parasympathetic tumors derived from the paraganglia and the adrenal medulla, respectively. Paragangliomas and pheochromocytomas can be sporadic or familial, the latter frequently being multifocal and possibly due to succinate dehydrogenase complex genes mutations. In addition, 12% of sporadic paragangliomas are related to covered succinate dehydrogenase complex mutations. The importance of identifying succinate dehydrogenase complex mutations is related to the risk for these patients of developing multiple tumors, including non-endocrine ones, showing an aggressive clinical presentation.

**Case presentation:**

We report the case of a 45-year-old Caucasian man with an indolent mass in his neck. Ultrasound of his neck, magnetic resonance imaging, and 1,4,7,10-tetraazacyclododecane-N(I),N(II),N(III),N(IIII)-tetraacetic acid(D)-Phe(1)-thy(3)-octreotide (^68^Ga-DOTATOC) positron emission tomography-computed tomography and endocrine work-up were consistent with a carotid body paraganglioma with concomitant nodal enlargement in several body regions, which turned out to be a follicular lymphoma at histology. He was found to carry a germline Succinate dehydrogenase subunit B gene (*SDHB*) mutation.

**Conclusion:**

It is crucial to look for a second malignancy in the case of a paraganglioma demonstrating succinate dehydrogenase complex germline mutations.

## Background

The terms paraganglioma (PGL) and pheochromocytoma (PHEO) refer to sympathetic or parasympathetic tumors arising from the paraganglia and the adrenal medulla, respectively [1–3]; PGL and PHEO together are named as PPGL. The annual incidence of PPGL is up to 3/million population [4]. Hereditary PPGLs represent 40% of the cases and are characterized by multifocal lesions in 35–50% of the patients [1, 3, 5, 6]. Succinate dehydrogenase (SDH) germline mutations represent a possible cause of hereditary PPGL, but have also been reported in 12% of sporadic cases [1]. Germline mutations in *SDHB*, *SDHC*, and *SDHD* genes have been found in PPGL, but also in gastrointestinal stromal tumors [2, 7]. Genetic screening of patients with SDH is crucial since hereditary forms are associated with a dismal prognosis and a high risk of developing other tumors [2, 3]. In particular, *SDHB* germline mutations show significant malignancy rates, which are higher than those found in patients with PPGL bearing different *SDH* germline mutations [8].

This case report describes the association of a neck PGL with a follicular lymphoma in a patient with a germline *SDHB* mutation.

## Case presentation

We report the case of a 45-year-old Caucasian man presenting with an indolent mass in his neck of 4 months’ duration, who was otherwise in good clinical condition. He did not take any prescribed medications and did not report any history of allergy, tobacco smoking, fever, sweating, headache, or hypertension. Familial anamnesis was negative. At a physical examination his thyroid appeared normal, while a subcutaneous 2 cm swelling of his neck was present at the superior margin of the right sternocleidomastoid muscle. The mass was indolent and of parenchymatous consistency at palpation, mobile with respect to the surrounding planes. Palpation also revealed a right submandibular 1.5 cm lymph node enlargement. Routine laboratory parameters were normal.

**Fig. 1:**
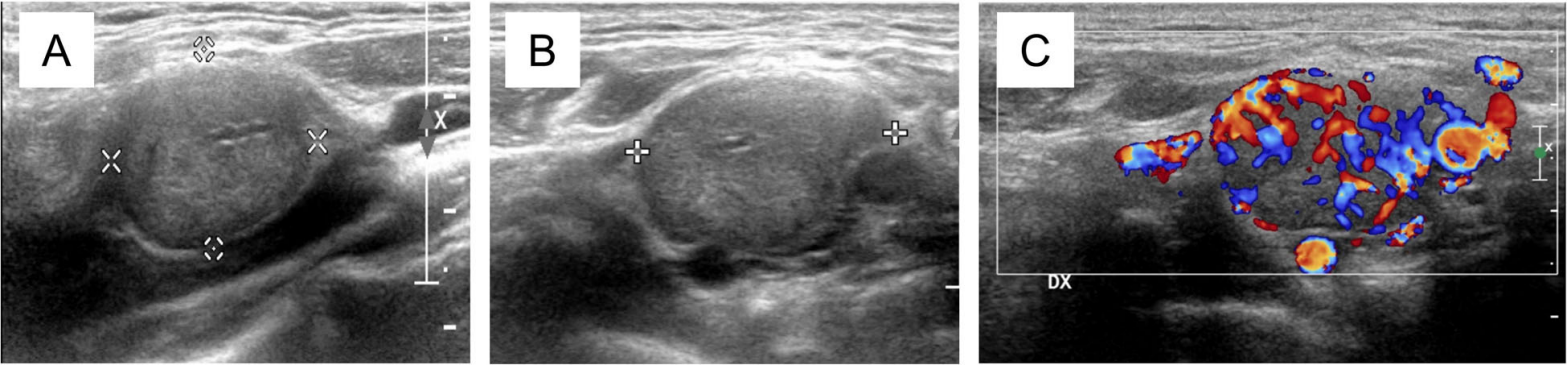
Neck ultrasonography (**a**) and **b** ultrasonography B-mode paraganglioma images; **c** axial color-Doppler ultrasonography image

He was subjected to ultrasonography (US) of his neck, which showed a 2.2 × 2.2 × 1.7 cm homogeneously hypoechoic oval lesion at the carotid bifurcation, with clear-cut margins, markedly vascularized at color-Doppler US. His thyroid had normal morphology and volume, while bilateral lymph node enlargement was observed at lateral cervical level II, devoid of characteristics suspicious for metastases (Fig. 1).

An endocrine work-up was requested, including urinary 24-hour catecholamines and metanephrines, plasma calcium, phosphate, parathyroid hormone, and chromogranin A; all were found to be in the normal range.

**Fig. 2:**
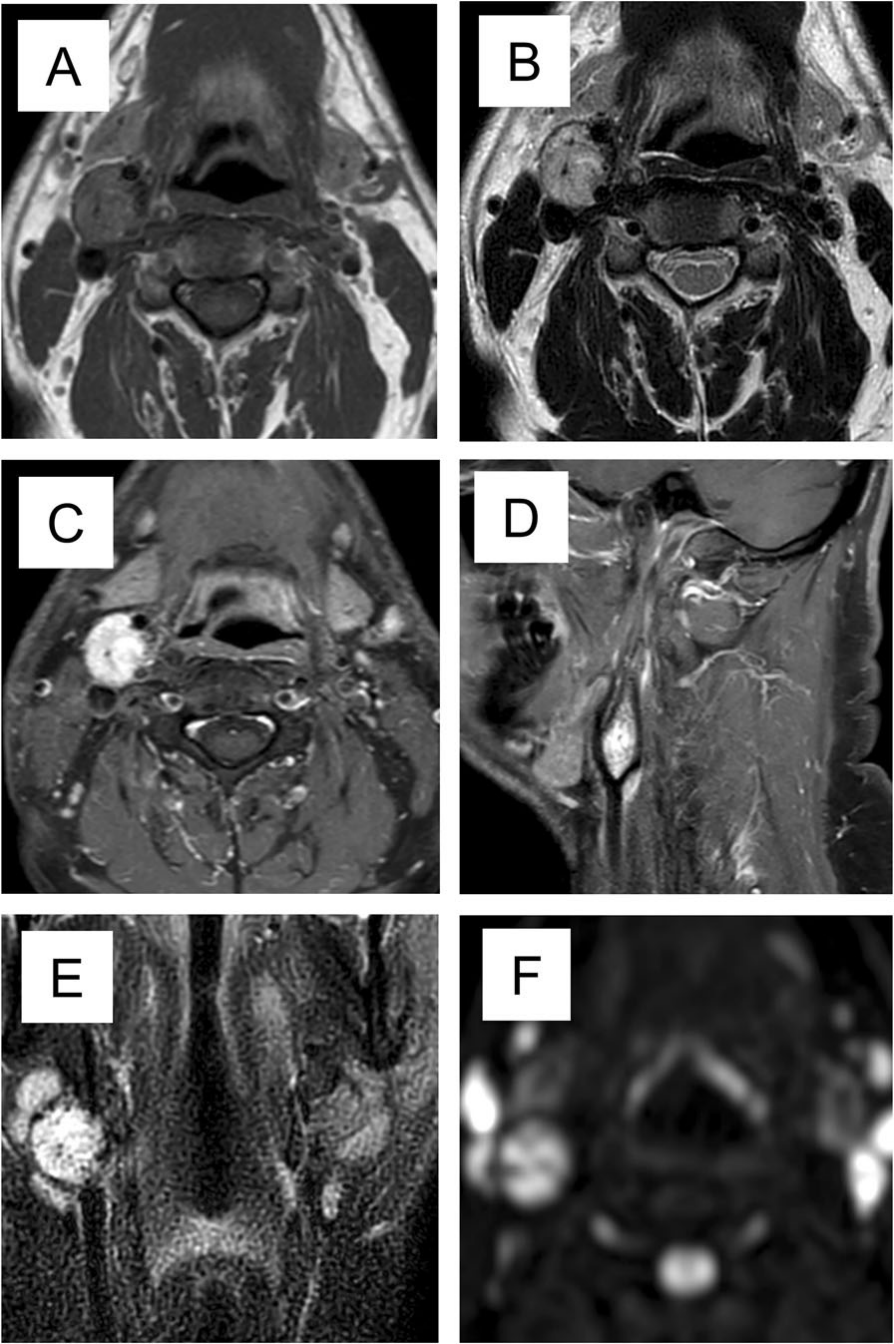
Neck magnetic resonance imaging. **a** Axial T1-weighted sequence; **b** axial T2-weighted image; **c**, **d** axial and sagittal images after contrast enhancement; **e** coronal short tau inversion recovery sequence; **f** diffusion-weighted sequence

He underwent magnetic resonance imaging (MRI) of his neck that showed a 2.3 × 2.5 cm neck mass, isointense to the muscular tissue on T1-weighted images (T1-wi) and hyperintense on T2-weighted images (T2-wi). After contrast administration the lesion showed marked vascularization and intralesional serpiginous areas. Several bilateral cervical lymph nodes were observed with axial maximum diameter of 1.2 × 0.8 cm and 1 × 0.9 cm at the I and II levels (Fig. 2).

**Fig. 3:**
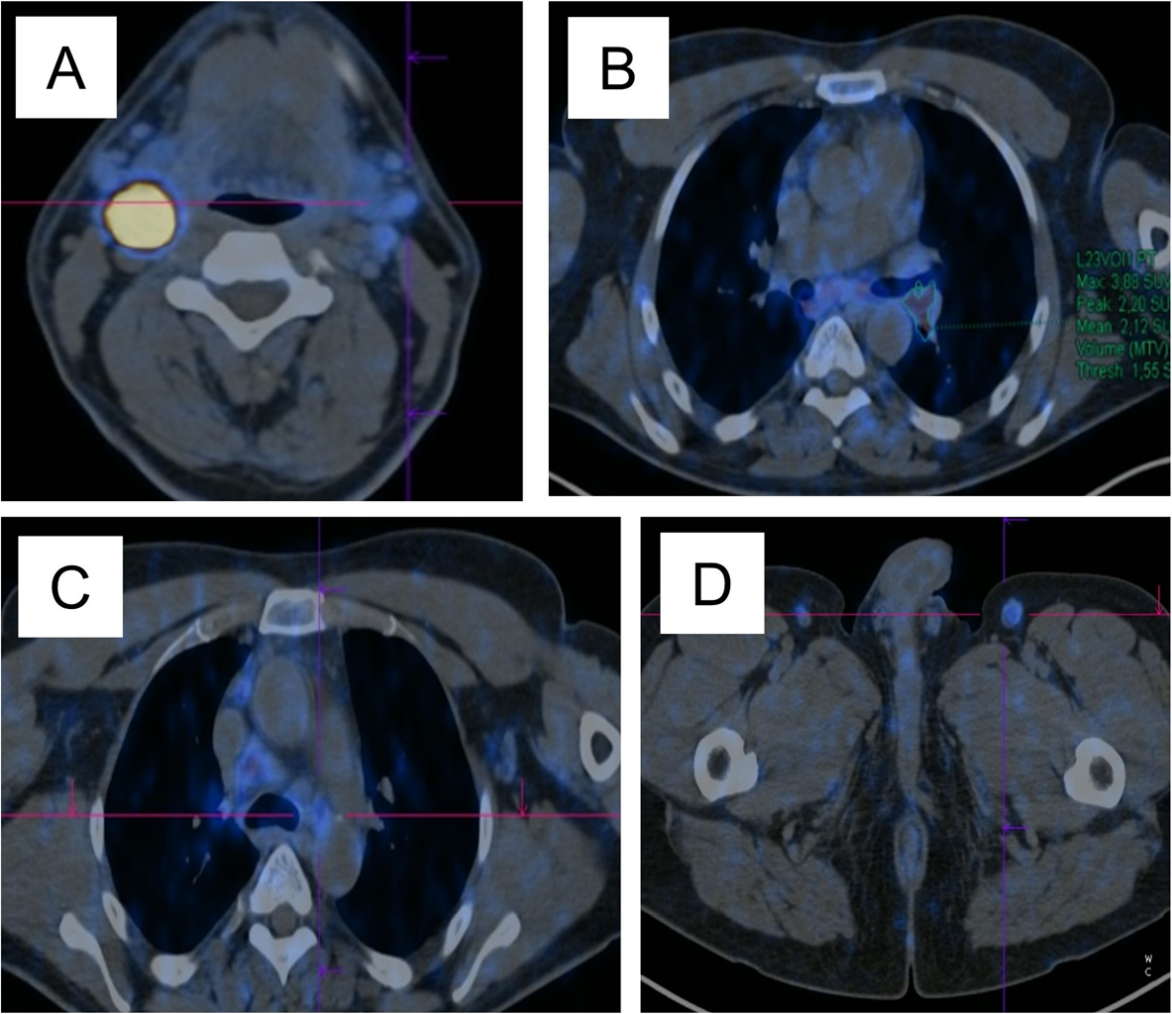
1,4,7,10-tetraazacyclododecane-N(I),N(II),N(III),N(IIII)-tetraacetic acid(D)-Phe(1)-thy(3)-octreotide (^68^Ga-DOTATOC) positron emission tomography/computed tomography axial fused images findings. **a** Intense focal uptake (maximum standardized uptake value 92.4) in the neck mass, strongly suggestive of paraganglioma; **b** hilar-mediastinal and peribronchial weak uptake (maximum standardized uptake value 3.9 in the pulmonary left hilum); **c** axillary and **d** inguinal weak uptake

On the basis of US and MRI, a PGL was suspected. A 1,4,7,10-tetraazacyclododecane-N(I),N(II),N(III),N(IIII)-tetraacetic acid(D)-Phe(1)-thy(3)-octreotide (^68^Ga-DOTATOC) positron emission tomography (PET)-computed tomography (CT) evaluation was performed, showing an intense focal uptake with a maximum standardized uptake value (SUVmax) of 92.4. Weak lymph node uptake was found in the following regions: lateral cervical, hilar-mediastinal, peribronchial, axillary, and inguinal stations, with a SUVmax of 3.9 in the pulmonary left hilum (Fig. 3).

According to current guidelines [3], our patient was submitted to genetic testing, including investigation for germline *RET* and succinate dehydrogenase complex (SDHx) mutations. Germline *RET* mutation testing was performed by direct sequencing as described before [9] and did not disclose any significant mutation. Germline SDHx mutation testing was performed on leukocyte DNA by a next-generation sequencing (NGS) method based on capture technology (probe; IDT), evaluating the presence of point mutations, small deletions, or insertion mutations in the coding region of the *SDHB*, *SDHA*, *SDHD*, *SDHAF2*, *SDHC* genes and flanking intronic regions by amplification and direct sequencing. The results of the genetic test of our patient showed the presence of a germline mutation in exon 7 of the *SDHB* gene. The sequence variant c.689>A; p.Arg230His in heterozygosis was confirmed in a second genomic DNA sample. This variant causes the replacement of the amino acid arginine with a histidine at *SDHB* protein codon 230. The arginine residue is highly conserved in the course of evolution. Moreover, this variant is present in the population database with a very low frequency, having been identified in one allele out of 251.416 studied. This variant is reported by multiple sources as causative, being classified as pathogenetic, and associated with PGL. The family history of our patient was negative for PPGL or other endocrine neoplasms.

Our patient underwent surgery and histology was consistent with a carotid body PGL. Due to the presence of a germline *SDHB* gene mutation, a second malignancy was suspected; therefore, accurate histology examination was also pursued for the neck lymph nodes resected during surgery. In fact, a follicular lymphoma was documented as grade 1 according to the histological classification of the World Health Organization (WHO). The hematological assessment of our patient followed European Society for Medical Oncology (ESMO) guidelines; therefore, a contrast-enhanced CT scan of his neck, thorax, abdomen, and pelvis with an ^18^FDG (^18^F-fluorodeoxyglucose)-PET examination established a stage IIIA in accordance with the Ann Arbor classification system [10]. A clinical follow-up with blood test (including a complete blood count, creatinine, uric acid, aspartate aminotransferase, lactate dehydrogenase, total protein, serum protein electrophoresis, and beta-2 microglobulin) and US examination at 3 months was performed, the results were normal. Chemotherapy was not deemed necessary.

## Discussion and conclusions

This case report underlines the importance of genetic screening in patients with PPGL, since second malignancies associated with hereditary forms are not rare. Most sporadic PGLs are benign and their prognosis is related to the tumor site: those arising at the carotid body have a better outcome as compared to those located at the skull base (jugular foramen, middle ear, and along the vagus nerve) [11]. A carotid PGL in a *SDHB* gene mutation carrier is rarely the first manifestation, since *SDHB*-related PGLs are usually found in extra-adrenal abdominal sites [8]. In fact, these tumors frequently produce high amounts of catecholamines, with associated PHEO-like clinical manifestations. By contrast, our patient showed a hypervascular, well-circumscribed, and asymptomatic enlarging soft tissue lesion in his neck, without adrenergic symptoms such as hypertension, headache, shaking, or weight loss. In our case, clinical manifestation was more consistent with a sporadic PGL that is mostly asymptomatic; when not, it may manifest clinically either as lateral cervical pulsatile mass or with symptoms (dysphagia, anisocoria, dysphonia, and deafness) due to compression and/or displacement of neighboring nerve structures [12]. Once again, our case underlines the importance of a correct genetic investigation, even in the absence of family history and of specific symptoms.

On the other hand, imaging was highly suggestive because in our patient the carotid body PGL appeared at US as a solid, hypoechoic, ovoid, well-defined mass, with a homogeneous structure; at Doppler US the mass appeared markedly hypervascular [13]. Similarly, MRI was highly indicative of PGL, since the tumor had a low to intermediate signal intensity on T1-wi and proton density-weighted sequences and a markedly high signal intensity on T2-wi. On the other hand, the pathognomonic “salt-and-pepper” pattern was not evident, in keeping with its inconstant presence [11].

Since a PGL was suspected, in the absence of catecholamine-related symptoms and of evidence of elevated urinary catecholamine levels, the choice for a ^68^Ga-DOTATOC PET/CT was appropriate, since ^123^I-metaiodobenzylguanidine (MIBG) PET or ^18^F-DOPA PET/CT may return false negative results [14]. On the other hand, ^68^Ga-DOTATOC PET/CT may identify lesions different from PGL and in this case showed multiple lymph node faint uptake sites. However, an US examination did not indicate a metastatic suspicion, and, together with ^68^Ga-DOTATOC PET/CT, pointed to the presence of inflammatory lymph nodes. On the basis of the results of the genetic testing and of the presence of abdominal ^68^Ga-DOTATOC PET/CT uptake, a second malignancy could not be ruled out, supporting the indication for lymphadenectomy during PGL surgery. In fact, a follicular lymphoma was discovered. It has been previously reported that follicular lymphomas express somatostatin receptors, in keeping with ^68^Ga-DOTATOC PET uptake in our case [15].

Among the reported malignancies associated with SDHx mutations, the presence of a malignant B cell lymphoma has been reported only in one Japanese patient bearing a G106D alteration in exon 4 of the *SDHD* gene, who developed the disease 5 years after PGL surgery [16]. In addition, genetic derangements in the *SDHD* gene have been found in other cases: a silent single nucleotide polymorphism was identified in three Burkitt’s lymphoma cell lines and in one Burkitt’s lymphoma sample [1, 17].

The association of a *SDHB* germline mutation with lymphoproliferative disorders, however, is not novel. Cases of *SDHB* carriers with T cell acute lymphoblastic leukemia [18] or Hodgkin lymphoma [19] have been reported. In addition, patients with PGL with synchronous or metachronous low-grade B cell non-Hodgkin lymphoma [20] and follicular lymphoma [21] have been described, but germline mutations were not investigated. Therefore, our case represents the first association between a *SDHB* germline mutation and a follicular lymphoma. To date, studies reporting a predisposition to lymphoid malignancies in patients with SDHx mutations are still lacking; also, SDHx genes are not routinely evaluated in individuals with non-endocrine cancers [19].

We conclude that in cases of hereditary PPGLs, other malignancies should be looked for, including hematological disorders.

## Data Availability

Data sharing is not applicable to this article, because no datasets were generated or analyzed during the current study.

## Abbreviations

^18^FDG: ^18^F-fluorodeoxyglucose
CT: Computed tomography
ESMO: European Society for Medical Oncology
MIBG: ^123^I-metaiodobenzylguanidine
MRI: Magnetic resonance imaging
NGS: Next-generation sequencing
PET: Positron emission tomography
PGL: Paraganglioma
PHEO: Pheochromocytoma
PPGLs: Paragangliomas and pheochromocytomas
SDH: Succinate dehydrogenase
SDHx: Succinate dehydrogenase complex
SUVmax: Maximum standardized uptake value
T1-wi: T1-weighted images
T2-wi: T2-weighted images
US: Ultrasonography
WHO: World Health Organization
